# Global Distribution of Anaerobic Ammonia Oxidation (Anammox) Bacteria – Field Surveys in Wetland, Dryland, Groundwater Aquifer and Snow

**DOI:** 10.3389/fmicb.2019.02583

**Published:** 2019-11-12

**Authors:** Yu Wang, Liya Xu, Shanyun Wang, Fei Ye, Guibing Zhu

**Affiliations:** ^1^Key Laboratory of Drinking Water Science and Technology, Research Center for Eco-Environmental Sciences, Chinese Academy of Sciences, Beijing, China; ^2^Institute of Environmental Research at Greater Bay, Key Laboratory for Water Quality and Conservation of the Pearl River Delta, Guangzhou University, Guangzhou, China

**Keywords:** anammox, nitrogen cycle, co-occurrence network, groundwater aquifer, global-scale, local-scale

## Abstract

The discovery of anaerobic ammonia oxidation (anammox) expanded our knowledge on the microbial nitrogen cycle. Previous studies report that anammox bacteria are distributed in a wide range of habitats and plays significant roles in the global nitrogen cycle. However, most studies focus only on individual ecosystems or datasets from public databases. To date, our understanding of how anammox bacteria respond to environmental properties and are distributed in different habitats on a global scale, remain unclear. To explore the global distribution of anammox bacteria, samples were collected from different habitats at different locations globally, including wetlands, drylands, groundwater aquifers and snow from 10 countries across six continents. We then used high-throughput amplicon sequencing targeting the functional gene hydrazine synthase (HZS) and generated community profiles. Results showed that *Candidatus Brocadia* is detected as the dominant genus on a global scale, accounting for 80.0% to 99.9% of the retrieved sequences in different habitats. The Jettenia-like sequences were the second most abundant group, accounting for no more than 19.9% of the retrieved sequences in all sites. The samples in drylands, wetlands and groundwater aquifers showed similar community composition and diversity, with the snow samples being the most different. Deterministic processes seem stronger in regulating the community composition of anammox bacteria, which is supported by the higher proportion explained by local-scale factors. Groundwater aquifers showed high gene abundance and the most complex co-occurrence network among the four habitat types, suggesting that it might be the preferred habitat of anammox bacteria. There is little competition between anammox bacterial species based on co-occurrence analysis. Hence, we could infer that environmental factors such as anaerobic and stable conditions, instead of substrate limitations, may be vital factors determining the anammox bacteria community. These results provide a better understanding of the global distribution of anammox bacteria and the ecological factors that affect their community structuring in diverse habitats.

## Introduction

The process of anammox (anaerobic ammonium oxidation), referring to the oxidation of ammonium coupled with the reduction of nitrite under anoxic conditions, leads to a loss of nitrogen in form of N_2_ gas (Strous et al., 1999; Jetten, 2001). The anammox bacteria is a deep-branching monophyletic group within the phylum *Planctomycetes* (Jetten et al., 2003). The roles of anammox bacteria in the nitrogen cycle have drawn extensive attention since it was first described in the 1990s (Mulder et al., 1995; Strous et al., 1999). A total of five genera have already been identified to date, including *Candidatus Brocadia*, Kuenenia, *Scalindua*, Anammoxoglobus, and Jettenia with 16 classified species (Sonthiphand et al., 2014). Anammox bacteria were proposed to be responsible for as much as 50% of the global removal of fixed nitrogen from the oceans (Dalsgaard et al., 2005). In freshwater lakes and agricultural soils, the anammox process was reported to account for up to 40 and 37% of the nitrogen loss, respectively (Yoshinaga et al., 2011; Zhu et al., 2011). These bacteria have also been detected in extreme environments such as hydrothermal vents and cold hydrocarbon-rich seeps (Russ et al., 2013). The distribution of anammox bacteria and their role in nitrogen loss are influenced by local environmental conditions: dissolved oxygen (Woebken et al., 2008; Lam and Kuypers, 2011), organic content (Thamdrup and Dalsgaard, 2002; Kalvelage et al., 2013), NO_x_^–^ (Risgaard-Petersen et al., 2004), environmental stability (Dalsgaard et al., 2005), and salinity (Dale et al., 2009) have been described as key factors. Succeeding studies showed that anammox based processes are more environmentally friendly compared to traditional nitrogen removal processes, as they mitigate greenhouse gas emissions of N_2_O and CO_2_ (Kartal et al., 2010; Wang et al., 2018). Until now, our understanding of the ecological functions and environmental significance of anammox bacteria is continues to grow.

In a recent study, anammox bacteria were resuscitated from a >10,000 years dormant state by the addition of water (Zhu et al., 2019). This finding suggests that the presence and distribution of anammox bacteria may be directly linked to their ecological functions. In light of global climate change, where increasing precipitation is expected, the dormant anammox bacteria present in different environments may also be revived, allowing them to be actively involved in global nitrogen cycles (Zhu et al., 2019). Anammox bacteria are highly habitat-specific and their community structure is largely dependent on prevailing environmental conditions (Dalsgaard et al., 2005; Dale et al., 2009; Zhu et al., 2015). For example, the anammox bacteria *Candidatus Scalindua* was almost exclusively only detected in marine environments (Schmid et al., 2007; Woebken et al., 2008; Lam and Kuypers, 2011). In contrast, the diversity of anammox bacteria in terrestrial and freshwater ecosystems is much higher, and the factors influencing their community structuring and interactions are presumably more complex. Specifically, organic carbon, ammonium, and nitrite were reported to be key factors in determining anammox bacterial community structures (Hu et al., 2012; Wang et al., 2012; Hou et al., 2013). However, to date, most studies done on annamox bacteria have only focused on individual ecosystems or datasets from public databases (e.g., GenBank). On the global scale, our understanding of how anammox bacteria respond to environmental factors and their distribution in different habitats, remains unclear.

The global distribution of microorganisms was first considered to be random (O’Malley, 2007) but recent evidence suggests otherwise (Martiny et al., 2006, 2011; Nelson et al., 2016). Deterministic and stochastic processes were suggested as the two basic mechanisms influencing microbial spatial community structuring (Martiny et al., 2006). Deterministic processes suggest that species are filtered out by local-scale factors (such as disturbance and nutrients level) and only occur at environmentally suitable locations (Leibold et al., 2004; Aguilar et al., 2014), while stochastic processes are driven by ecological drift and affected by regional- scale factors (such as climate conditions and migration) (Martiny et al., 2011; Zhou et al., 2014a). In natural ecosystems, the two processes concurrently act in structuring the assembly of microbial communities, but many environmental parameters seem to be of particular importance in different systems (Zhou et al., 2014a). For anammox bacteria, how multiple environmental factors interact to determine their distribution on a global scale, remains unknown and is critical for understanding the habitat preference and for predicting their potential ecological functions in the global nitrogen cycle.

In this study, field surveys of anammox bacteria were conducted, covering different habitats and locations, but excluding the marine environment due to its expected low diversity (Schmid et al., 2007; Woebken et al., 2008; Lam and Kuypers, 2011). As one of the most wide-ranging studies on the spatial distribution of anammox bacteria to date, this work attempts to address the following questions: (i) What is the global distribution pattern of anammox bacteria? (ii) How do the habitats affect community structuring of anammox bacteria? (iii) What is the most suitable habitat for anammox bacteria and the underlying mechanism of the habitat preference?

## Materials and Methods

### Study Sites

**FIGURE 1:**
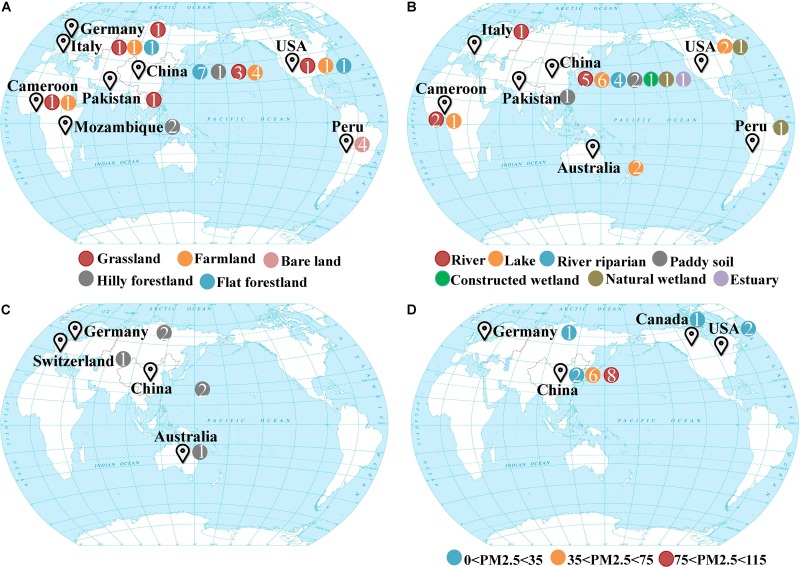
Global maps showing the sampling sites of four different ecosystems **(A)** drylands, **(B)** wetlands, **(C)** groundwater aquifers, **(D)** snow. Colors indicate various habitats where samples were taken from, and *n* indicates the sample number.

Samples were collected from four different systems in the biosphere, which included drylands, wetlands, groundwater, and snow. Drylands and wetlands represent two of the most active and contrasting ecosystems on earth’s surface in terms of biogeochemistry. Groundwater and snow represent habitats beneath and above the earth’s surface, respectively. The sampling sites representing these different ecosystems were distributed over 10 countries and six continents around the world (Figure 1; Supplementary Tables S1, S2). The dryland ecosystem habitat types included grasslands, forestlands, agricultural farmlands, and bare land (unutilized land, covered by sparse grass or no plants in some seasons). The sediment samples from wetland ecosystems included freshwater rivers, lakes, riparian zones, paddy fields, estuaries, natural, and constructed wetlands. Fresh snow was collected at various levels of air quality according to the PM2.5 values (25 to 115 μg/m3) which refers to atmospheric particulate matter that has a diameter of less than 2.5 μm at sampling time. All the samples were collected from October 2015 to January 2016, totaling 89 samples.

The samples were processed for chemical analysis according to previous studies (Zhu et al., 2013; Zhou et al., 2014b). In brief, for each sampling site, three soil/sediment samples were collected parallel to each other from three plots (10 × 10 m). In each soil plot, samples of the soil organic layer (10 × 10 cm in area, and 0–5 cm depth) were collected at three random points using a sterile blade or auger (Ø 2 cm) and mixed together in a sterile plastic bag as a single sample. For each groundwater aquifer soil/sediment sample, three large soil pits (100 × 100 cm, 9 m apart) were dug in an approximately 100 m^2^ sized area using PowerProbe and GeoProbe direct push drill rigs, and three parallel samples were obtained at each sampled layer (Zhu et al., 2013, 2018a,b). Soil pH was measured after shaking with water at a soil/water ratio of 1:5 (weight/volume). Soil moisture content (MC) was measured gravimetrically by oven-drying at 105°C for 48 h (Ye et al., 2019). NH_4_^+^, NO_2_^–^, and NO_3_^–^ were determined by flow injection analysis (FIA Star 5000, FOSS Tecator, Sweden) after extraction with 2 M KCl solution at a soil/KCl ratio of 1:5 (weight/volume) (Ye et al., 2019). Soil total organic matter (TOM) was measured through LOI_550_ (Loss on ignition at 550°C) (Dean, 1974). The content of TC, TN, and TS was measured by an Element Analyzer (Vario EL cube, Elementar, Germany) (Wang et al., 2019). For snow samples, we collected the fresh snow using a sterile plastic sheet placed on the ground when the snow began to fall. At each snow sample point (100 × 100 m), the four corners and the center position of the square were sampled as five independent replicates, which were mixed into one sample. Once collected, visible roots and residues were removed prior to homogenizing the soil/sediment fraction of each sample. Part of the soil/sediment samples were freeze-dried, and snow (after melting) samples were melted and immediately filtered through a 0.22 μm filter paper (Millipore, MA, United States) for bacterial DNA. Samples were stored in a cooler and then transported to the laboratory for further processing.

After checking by PCR amplification targeting the anammox bacterial specific gene, 74 samples (dryland = 31, wetland = 31, groundwater aquifer = 6, fresh snow = 6) tested positive for annamox bacterial gene and were then kept for downstream analyses.

### DNA Extraction and PCR Amplification

Genomic DNA from soil and sediment samples were extracted from ∼ 0.33 g subsamples after freeze drying. The DNA from snow samples was extracted from 300–500 ml thawed snow water after being filtered through a 0.22 μm filter. DNA was extracted with a Fast DNA SPIN Kit for Soil (MP Biomedicals, United States), quantified and assessed for quality using a Nano Drop 2000 UV-Vis Spectrophotometer (Thermo Fisher Scientific, United States). PCR amplification of the anammox bacteria hydrazine synthase (HZS) was done using the hzsB_396F and hzsB_742R primer pairs (Wang et al., 2012). Amplification was performed in 50 μl reaction mixtures containing 5 μl 10 × buffer, 4 μl dNTP (2.5 mmol/L), 1 μl of each primer (10 mmol/L), 0.5 μl BSA, 0.25 μl Taq polymerase (2.5 U) (Takara, Japan) and 2 μl of the DNA template, and dd H_2_O made up to 50 μl. The amplification thermal protocol consisted of following steps: 10 min at 95°C, then 35 cycles of 60 s at 95°C, followed by 60 s at 59°C, and 45 s at 72°C, and an extension of 10 min at 72°C. The PCR products were purified in 1% agarose gel after electrophoresis using the Wizard SV Gel & PCR clean-up system (Promega, United States) before multiplexing sequencing using the Hiseq 2500 platform (Illumina, United States).

### Quantitative PCR

The abundance of anammox bacteria was determined by quantitative PCR using primer sets of hzsB_396F and hzsB_742R (Wang et al., 2012). Amplification was performed in 20 μl reaction mixtures: 10 μl SYBRs Premix Ex Taq (Takara, Japan), 0.4 μl ROX50, 0.2 μl of each primer (200 nmol/L), 0.4 μl bovine serum albumin (BSA) and 2 μl of the DNA template, and ddH_2_O added to 20 μl. The ddH_2_O was used as the template of the negative control. The DNA of soil samples that had been identified to have anammox activity were used as templates of the positive controls (Wang et al., 2019). The thermal cycles included 3 min at 95°C, followed by 40 cycles of 30 s at 95°C, 30 s at 59°C, and 30 s at 72°C. Standard curves were generated from 10-fold serial dilutions of the plasmid DNA. The results with an efficiency and correlation coefficient above 90% and 0.95 were utilized.

### High-Throughput Sequence Processing

Raw paired-end reads of nucleic acids were assigned to samples based on their unique barcodes, which were later truncated with the primer sequences. Specifically, chimeric sequences produced during incomplete PCR amplification were deleted using the Uchime algorithm (V1.7.0) (Caporaso et al., 2010). Then low-quality sequences were removed using the QIIME. Redundant nucleic acid sequences were then determined using Mothur (Schloss et al., 2009), followed by translation into amino acid sequences using the BioEdit software (Version 7.2). Sequences demonstrating 90% similarity were assigned to the same operational taxonomic unit (OTU). Representative sequences were assigned with taxonomy using the Basic Local Alignment Search Tool (BLAST) and compared against the *hzs*B gene sequences from the NCBI database. Raw data of the high throughput sequencing were deposited in the NCBI Sequence Read Archive (SRA)^1^ under accession number SRX3003873.

### Statistical and Network Analysis

The Mann–Whitney *U* test was used to assess significant differences in anammox bacterial α-diversity indices (i.e., Shannon diversity, Chao1 richness, and Pielou evenness) between and among different habitats (IBM SPSS Statistics 20.0 for Windows), as well as non-metric multidimensional scaling (NMDS) using Bray-Curtis dissimilarity of anammox bacterial OTUs and α-diversity. Variation partitioning analysis (VPA) was then used to quantify the relative influences of environmental factors on the anammox bacterial community in CANOCO 5 software. Analysis of similarity (ANOSIM) was calculated at the OTU level to infer the dissimilarity of the anammox bacterial community between and among habitats using the R platform with the vegan package (Version 3.0.1, permutations = 999). Spearman’s rank correlation between the abundance of anammox bacteria and environmental variables was plotted using the “corrplot” package in R (version 3.0.1).

A correlation-based network analysis was carried out to explore the co-occurrence patterns of anammox bacteria in different habitats. To visualize the associations in networks, a correlation matrix was constructed by calculating for pairwise Spearman’s rank correlations based on the OTU level. The Spearman’s rank correlation coefficient (ρ) absolute value >0.8 and the *P*-value < 0.05 were considered to be a statistically robust correlation between OTUs. The nodes and edges in the networks correspond to the anammox bacterial taxa (OTUs) and the strong correlations between the nodes, respectively. Topological characteristics of the networks were calculated to describe the complex pattern of interrelationships among anammox bacterial OTUs. The Spearman’s rank correlations were calculated with Python 2.6, and networks were visualized using Gephi software (Bastian et al., 2009).

## Results

### Global Distribution of Anammox Bacterial Community Composition

After filtering out sequences of incongruent lengths and low quality, a total of 975,093 reads were retained for further analyses (Supplementary Table S3). Blasted results in the GenBank database showed that the majority of the processed sequences were highly similar to the anammox bacterial *hzs*B gene. Most of them belonged to *Candidatus Brocadia*, *Candidatus Jettenia*, *Candidatus Kuenenia*, and *Candidatus Scalindua*. Sequences that were distantly related to these genera were defined as “*unknown”* in a subsequent analysis.

**FIGURE 2:**
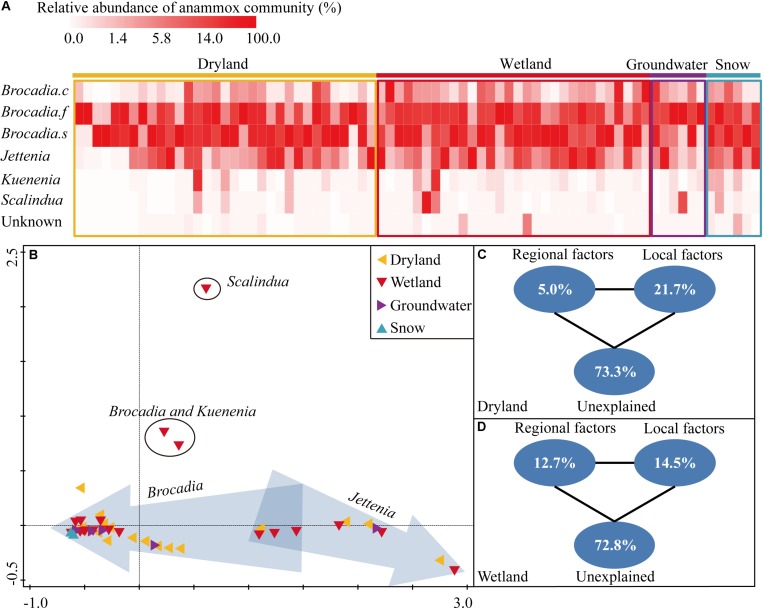
Heatmap showing the relative abundance of each species or genus in each sampling site **(A)**, the non-metric multidimensional scaling ordination (NMDS) based on Bray-Curtis similarity of anammox bacterial communities at each sampling site. Sites in arrows indicated that the dominant genus was Jettenia or Brocadia **(B)**, variation partitioning analysis showing the relative influence of environmental factors on the structuring of the anammox bacterial community in drylands **(C)** and wetlands **(D)**.

Globally, *Candidatus* Brocadia was predominant in all four systems, accounting for 80.6%, 77.9%, 80.0%, and 99.9% of the retrieved sequences in drylands, wetlands, groundwater aquifers and snow habitats, respectively (Figure 2A). Within *Candidatus Brocadia*, *Brocadia fulgida*, and *Brocadia sinica* related sequences accounted for as much as 23.8–52.7% and 41.2–55.8% of the total sequences, which were the dominant species. The *Candidatus* Jettenia was the second most abundant genus which composed 18.4%, 16.8%, and 19.9% of the retrieved sequences in the dryland, wetland and groundwater aquifer systems, respectively. No Jettenia-like sequence was detected in the snow samples. The Kuenenia-like sequences only accounted for a very small fraction in each habitat (0–2.6%). Similarly, the Scalindua-like sequences were only 0–2.8% of the sequences, with the highest abundance in a wetland site.

The community composition of sites in drylands and wetlands, revealed by the *hzsB* gene, showed considerably high variation. For instance, the majority of sites in both drylands and wetlands clustered together (Figure 2B), and were dominated by *Candidatus* Brocadia. Another three dryland sites and three wetland sites clustered together, and were dominated by *Candidatus* Jettenia. In another cluster, which included three wetland sites and two dryland sites, the Brocadia- and Jettenia-like sequences were of comparable proportions. In contrast, a relatively low variation in the community composition was observed on the snow samples sites. Notably, one sample from a riparian sediment of a city river was dominated by Scalindua-like sequences which are frequently detected in marine environments in previous studies.

The role of regional-scale factors (e.g., average annual temperature, precipitation, and sunshine duration) and local-scale factors (e.g., soil/sediment physicochemical properties) in the structuring of the anammox community were quantified by a variation partition analysis (VPA). In drylands, the two type of factors explained 26.7% of the observed variation, while local and regional factors independently explained 21.7% and 5.0% of the variation, respectively (Figure 2C). In wetlands, the local factors explained more variation than the regional factors (14.5% versus 12.7%), leaving 72.8% of the variance unexplained. But effects of the local factors were not as strong as in the drylands (14.5% versus 21.7%) (Figure 2D). Samples in groundwater aquifers and snow were excluded in the VPA due to their relatively low sample numbers.

### Abundance of Anammox Bacteria

**FIGURE 3:**
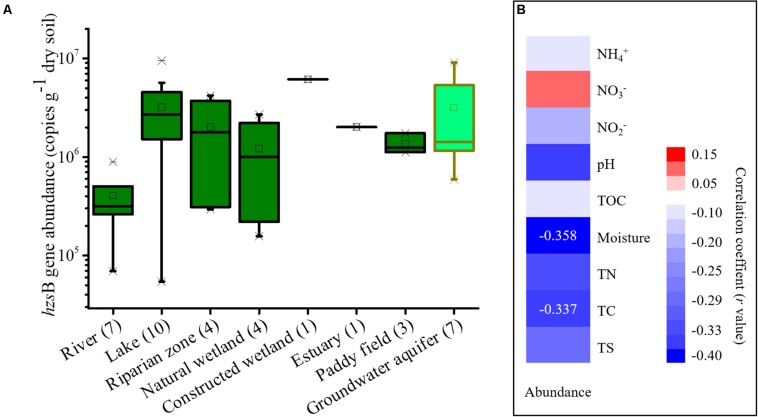
Boxplots showing the abundance of the *hzs*B gene in each wetland habitat and groundwater aquifer. **(A)** and a correlation analysis of anammox bacterial abundance and chemical parameters **(B)**. The dryland and snow samples failed to be quantified by qPCR assay due to their extremely low abundance. In this figure mean values are indicated by the open square, the central horizontal bars are the medians, and the box lower and upper limits are the 1st and 3rd quartiles, respectively. Crosses are minimum/maximum values. Sample numbers are indicated after the habitat name.

The gene abundance of anammox bacteria in wetlands revealed by the *hzsB* gene ranged from 5.42 × 10^4^ to 9.56 × 10^6^ copies/g of dry soil (Figure 3A). Among these habitats, sediments from freshwater rivers showed a low abundance (6.9 × 10^4^ to 8.9 × 10^5^ copies/g of dry soil). In contrast, freshwater lakes showed a higher abundance at 5.4 × 10^4^ to 9.6 × 10^6^ copies/g of dry soil. It was noted that the abundance of anammox bacteria in groundwater aquifers was high, ranging from 5.93 × 10^5^ to 9.12 × 10^6^ copies/g of dry soil. A correlation analysis suggested that the chemical conditions had lower to no effect on the abundance of these samples and that only soil moisture content (*P* = 0.035) and total carbon (*P* = 0.048) showed significant correlations with their abundance (Figure 3B). In comparison, the samples from drylands and snow failed to be quantified by the qPCR assay due to their extremely low abundance.

### Alpha-Diversity of Anammox Bacteria

**FIGURE 4:**
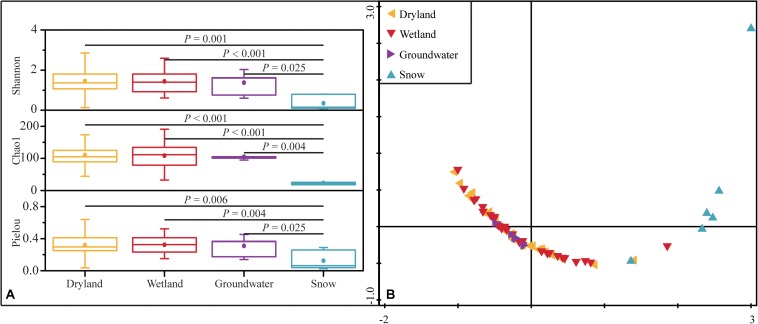
Alpha-diversity represented by Shannon, Chao1 and Pielou indices in the four ecosystems. The *P*-values indicate differences between groups based on the Mann–Whitney *U* test **(A)**, and NMDS analysis of α-diversity based on Euclidean distances of the four diversity indices (Shannon, Simpson, Pielou and Chao1) of the anammox bacterial community **(B)**.

Shannon diversity, Chao1 richness, and Pielou evenness were calculated to determine α-diversity according to the retrieved *hzsB* sequences. Drylands, wetlands, and groundwater aquifers showed comparable levels of diversity based on these indices. The Shannon index in these three types of habitats were 0.13–3.26, 0.61–2.59, and 0.60–2.03, the Chao1 indices were 44–186, 33–191, and 95–120, respectively (Figure 4). The Pielou indices were 0.04–0.64, 0.15–0.52, and 0.14–0.45 in drylands, wetlands, and groundwater aquifers, respectively. In contrast to their extremely low abundance, the alpha-diversity in drylands was high. However, snow had the lowest diversity among the samples (*P* < 0.01, one-way ANOVA). Such observations were confirmed by the NMDS, where samples in drylands, wetlands, and groundwater aquifers clustered closer together, while the snow samples were farther distributed.

### Co-occurrence Patterns in Anammox Bacterial Community

**FIGURE 5:**
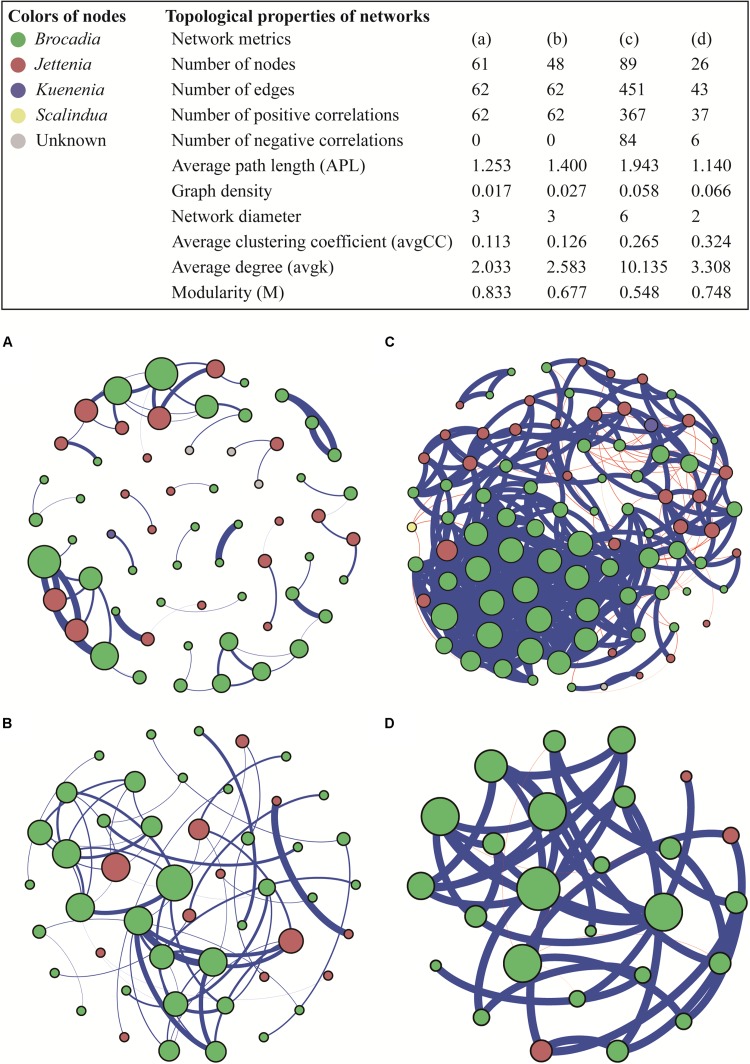
Ecological networks of co-occurring anammox bacterial OTUs in drylands **(A)**, wetlands **(B)**, groundwater aquifers **(C)** and snow **(D)**, based on correlations. A connection corresponds to a strong (Spearman’s ρ > 0.8) and significant (*P* < 0.05) correlation. The size of each node is proportional to the number of connections (i.e., degree); the thickness of each connection between two nodes (i.e., edge) is proportional to the value of Spearman’s correlation coefficients. A blue edge indicates a positive interaction between two individual nodes, while a red edge indicates a negative interaction.

The co-occurrence patterns of anammox bacteria were explored using a network analysis based on strong and significant correlations. The number of positive correlations was much higher than the negative correlations in all four networks (ρ > 0.8, *P* < 0.05) (Figure 5). Drylands and wetlands showed similar co-occurrence patterns with comparable numbers of positive correlation, negative correlation, and edges. However, the ecological network in groundwater aquifers was markedly different in terms of the number of nodes, edges, and significant correlations, which were considerably higher than the other three ecosystems. Furthermore, the average path length (APL), indicating the average network distance between all pairs of nodes, showed the highest value in groundwater aquifers, while the network in the snow sample was the simplest with the lowest number of nodes, edges, and significant correlations. The average clustering coefficient (avgCC), which showed that the nodes tend to cluster together, ranged from 0.113 to 0.324 in the four networks, and the groundwater aquifers and snow showed relative higher values. A similar trend was also observed in the graph density, with higher values in groundwater aquifers and snow.

## Discussion

This study revealed that anammox bacteria were widespread in different ecosystems, covering a wide range of habitats and geographical regions. To our knowledge, this is the largest field investigation in terms of scale on anammox bacteria, focusing on their distribution and community structure, to date. Applying similar procedures of sampling and molecular analysis in one study allowed us to avoid experimental biases to compare results.

Globally, the *Candidatus* Brocadia was the most dominant genera of anammox bacteria in natural ecosystems, followed by *Candidatus* Jettenia. It is consistent with previous studies showing that the *Brocadia*, *Kuenenia*, and *Jettenia* are the most common representatives in terrestrial and freshwater systems (Humbert et al., 2010; Zhu et al., 2011; Wang et al., 2012; Bai et al., 2015). The dominance of *Candidatus Brocadia* is mostly associated with their versatile metabolism (e.g., multiple physiological capabilities and environmental tolerance), allowing them to be more adaptable to various environments (Gori et al., 2011).

On the global scale, local-scale factors associated with soil/sediment characteristics were stronger in explaining the variation in the anammox bacterial community. In contrast, regional-scale factors such as temperature, precipitation, and the duration of sunlight plays a lesser role, implying a stronger role of the deterministic process. Similar conclusions, that the global pattern of bacterial community structures is related to local factors such as the nutrient level (Demir-Hilton et al., 2011) and soil moisture (DeBruyn et al., 2011) have been suggested. This is distinct from the global distribution of plants, in which temperature and precipitation are the key factors in shaping a community structure (Takenaka, 2005). This indicates that the growth of plants and microorganisms are generally limited by different factors. For the bacteria of the anammox group, the micro-environment in the habitat is more important.

Local factors explain more variation in the drylands compared to the wetlands. It is probably because soil is more heterogeneous in terms of moisture content, nutrient level and other factors, which could diversify the species’ coexistence and composition (Reynolds et al., 1997). In aquatic systems, however, water might potentially sweep the local environmental gradients, decreasing the effects of local factors (Xiong et al., 2017). Moreover, the presence of water in wetlands satisfied the basic demands of the anammox bacteria, either by creating an anaerobic environment or by carrying the substrates for anammox activity (Zhou et al., 2014b). Therefore, other local factors related to soil properties in wetlands is not as important as in drylands.

Anammox bacteria was also unexpectedly detected in snow samples, with a distinct community composition and diversity. This suggests that anammox bacteria is a component of an airborne bacterial community which could come from biological aerosols like other microorganisms (Morris et al., 2011). However, it remains inconclusive that the atmosphere represented by snow was another habitat for anammox bacteria, in addition to their well-known habitats (i.e., drylands, wetlands, groundwater aquifers and the ocean), since anammox bacteria have strict requirements for a stable and anaerobic environment (Strous et al., 1998; Jetten, 2001; Dalsgaard et al., 2005; Wenk et al., 2013). Moreover, the doubling time of anammox bacteria in the best growth conditions needs at least 10–12 days (van der Star et al., 2007). However, the life span of atmospheric particulates is less than 6 weeks, even for fine particles (PM2.5) (Steinfeld, 1998), which is neither long nor stable enough to support the proliferation of anammox bacteria. Hence, anammox bacteria may merely attach to soil particles and disperse with air flow.

The long-range dispersal of microorganisms including pathogens, algae, and bacteria along with soil particles have been long observed (Griffin et al., 2001). During transport, dust particles can serve as vessels for their global dispersion (Griffin et al., 2003). It has been estimated approximately 2 × 10^9^ metric tons of soil or sediment (dried river and lakebeds) is transported in the planet’s atmosphere each year (Perkins, 2001). In this scenario, anammox bacteria could disperse over very long distances, up to 1000s km (Steinfeld, 1998), by attaching to atmospheric particulates. Transport *via* atmospheric particulates is probably one of the many paths facilitating the dispersal and the worldwide distribution of anammox bacteria. The presence of anammox in aerial particles could somewhat explain why anammox bacteria could be detected in some environments using molecular approaches but tend to be inactive by isotope probing (Zhu et al., 2015).

Among the four types of habitats, anammox bacteria preferred groundwater aquifers more, which was evidenced by two aspects: a high abundance and the most complex community network. The samples in groundwater aquifers showed high gene abundance compared to those in the drylands, snow and some sites in wetlands. In the complex bacterial communities, abundance was a key indicator of their functional role (Rivett and Bell, 2018). The high anammox bacterial abundance observed here, may therefore be indicative of high anammox rates, which aligns with previous work where anammox was shown to be the dominant nitrogen loss pathway in unconfined aquifer soils (Wang et al., 2017). It has been identified that anammox is the dominant nitrogen loss pathway in unconfined aquifer soils (Wang et al., 2017). On the other hand, co-occurrence patterns in the groundwater aquifer showed the highest number of nodes, edges, positive correlations and average degree. These topological properties suggest that anammox bacteria in the groundwater aquifer had a complex and closely related community structure, which would increase the stability of interaction networks and enhance the resistance of the community to environmental disturbance (Krause et al., 2003).

A co-occurrence network analysis, which usefully represents various biological interactions in an ecosystem (Layeghifard et al., 2017), was conducted in this study. Notably, positive correlations dominated negative correlations in all four habitat types. In a network, positive interactions suggest that species are associated by commensalism or mutualisms, while negative interactions lead to predation and competition relationships (Chow et al., 2013). In this study, the low number of negative correlations implied that the competition between different species of anammox bacteria was weak in the four ecosystems. Inter-species competition, which is a major factor structuring species’ communities, is often emphasized as facing limited resources (Schoener, 1983; Vitousek and Howarth, 1991). Therefore, the low level of competition suggested that the presence and structure of the anammox community was probably not regulated by the availability of nutrient resources.

Although nutrient concentrations remained elevated at most of our sampling sites, which were greater than 0.17 mg.kg^–1^ (25.51 μM) for NH_4_^+^ and 0.02 mg.kg^–1^ (1.17 μM) for NO_2_^–^ (Supplementary Tables S1, S2), we cannot rule out the possibility of nutrient competition between anammox and competitors in some micro-habitats. Anammox bacteria were identified to have high substrate affinities, with half-saturation constants (K_s_) as low as 7 μM for NH_4_^+^ (Jetten et al., 2005) and 5 μM (*Brocadia*) and 0.2–0.3 μM (*Kuenenia*) for NO_2_^–^ (Kartal et al., 2012). Whereas, the K_s_ (NH_4_^+^/NH_3_) of ammonium oxidizing bacteria were higher than 10 μM (*Nitrosomonas*) and the K_s_ (NO_2_^–^) of nitrite-oxidizing bacteria of *Nitrospira* was 10 μM and 21–135 μM of *Nitrobacter* (Blackburne et al., 2007). Therefore, anammox bacteria would be a stronger competitor for NH_4_^+^ and NO_2_^–^ compared to most of their competitors, with substrate availability not influencing anammox bacterial community. Instead, environmental stress like oxygen exposure and habitat stability are more likely to be key factors.

It is well acknowledged that groundwater is the world’s largest freshwater resource, and it is also an extremely stable habitat. The relatively low influx in groundwater systems with a residence time of about 1,400 years, which is 70 times longer than that of surface waters (rivers, lakes, or wetlands) (Aeschbach-Hertig and Gleeson, 2012), suggests that the groundwater habitat is generally more stable compared to surface water bodies. Therefore, in addition to sufficient substrates for the anammox process (mostly NO_3_^–^), the stable environment, which is essential for anammox bacteria (Dalsgaard et al., 2005), would greatly facilitate its growth in groundwater. This could explain why the preferred habitat for anammox bacteria was the groundwater aquifer.

## Conclusion

Anammox bacteria were widely detected in the biosphere, including different habitats on earth’s surface (e.g., grasslands, forestlands, agriculture drylands, freshwater rivers, lakes, paddy fields, and wetlands), as well as beneath and above the earth’s surface (e.g., groundwater aquifers and the snow). Globally, the *Candidatus Brocadia* dominates the community, followed by *Candidatus Jettenia*. The habitat systems of drylands, wetlands, and groundwater aquifers showed similar patterns of community composition and diversity of anammox bacteria. However, the snow samples were significantly different from the other three systems. Local-scale factors were stronger in explaining the variation of community composition, implying that the global distribution of anammox bacteria were more likely to follow a deterministic process. Groundwater was identified to be a preferred habitat for anammox bacteria based on their high gene abundance and the complex co-occurrence network. Low competition was observed between species, implying that substrate availability (NH_4_^+^ and NO_2_^–^) is not a regulator for the anammox community, instead, environmental pressures such as oxygen exposure and unstable habitat conditions may play more vital roles.

## Data Availability Statement

The datasets generated for this study can be found in NCBI Sequence Read Archive, SRX3003873.

## Author Contributions

GZ and YW conceived the research. LX and SW performed the experiments. YW wrote the manuscript. GZ, YW, LX, SW, and FY edited the manuscript. LX and FY contributed to the data analysis and graphics pipeline. All authors reviewed and accepted the manuscript.

## Conflict of Interest

The authors declare that the research was conducted in the absence of any commercial or financial relationships that could be construed as a potential conflict of interest.

## Footnotes

1 http://trace.nvbi.nlm.nih.gov/Traces/sra/sra.cgi
