# Supplementary material for: Global Distribution of Anaerobic Ammonia Oxidation (Anammox) Bacteria – Field Surveys in Wetland, Dryland, Groundwater Aquifer and Snow
Source: Front Microbiol. 2019 Nov 12;10:2583. doi: 10.3389/fmicb.2019.02583 (PMC6861858; doi:10.3389/fmicb.2019.02583)
Supplement: Supplementary file 1 [file Data_Sheet_1.pdf]

Supplementary Table 1 Sampling sites and background information in drylands & wetlands.

Country		Coordinate	Habitat Types	regional-scale factors			local-scale factors								
DRYLAND				PRE (mm $year^{-1}$)	T (°C $year^{-1}$)	SD (h year $year^{-1}$)	NH_4^+ (mg kg^{-1})	NO_3^- (mg kg^{-1})	NO_2^- (mg kg^{-1})	pH	TOM (%)	MC (%)	TN (g kg^{-1})	TC (g kg^{-1})	TS (g kg^{-1})
1	Cameroon	4°37'N, 9°28'E	grassland	2000	24	1825	1.28	0.03	0.62	4.09	0.07	13.3	2.23	22.61	0.46
2	Cameroon	4°37'N, 9°28'E	farmland	2000	24	1825	5.41	0.82	0.03	3.6	4.35	11.65	1.12	14.79	0.39
3	Peru	12°06'S, 76°55'W	bare land	1000	19	3800	1.77	11.32	0.55	7.66	0.02	16.33	0.79	11.18	7.23
4	Peru	12°06'S, 76°55'W	bare land	1000	19	3800	1.08	9.06	0.63	7.36	0.03	12.51	0.29	2.62	0.88
5	Peru	12°06'S, 76°55'W	bare land	1000	19	3800	5.22	0.15	0.52	5.06	0.08	16.02	0.34	3.4	2.4
6	Peru	12°06'S, 76°55'W	bare land	1000	19	3800	1.83	3.46	0.6	7.32	0.04	15.75	0.91	14.56	0.6
7	Mozambique	18°48'S, 32°47'E	hilly forestland	1200	21	2920	0.17	9.64	0.53	4.34	0.22	15.11	4.89	73.93	0.68
8	Mozambique	18°48'S, 32°47'E	hilly forestland	1200	21	2920	0.3	2.18	0.44	4.29	0.08	8.54	1.36	23.89	0.22
9	China	24°26'N, 118°04'E	farmland	1743	22	1722	1.58	15.87	0.37	5.38	69.7	11.63	2.63	44.4	0.71

10	China	24°26'N, 118°04'E	flat forestland	1743	22	1722	5.38	1.28	0.16	5.07	14.7	13.15	3.13	40.57	0.23
11	China	29°30'N, 106°30'E	farmland	1246	17	1304	6.04	11.2	0.1	4.82	48.1	14.12	2.32	22.38	0.76
12	China	29°30'N, 106°30'E	hilly forestland	1246	17	1304	1.37	17.56	0.13	5.2	3.75	13.45	0.84	23.07	0.55
13	China	29°36'N, 106°42'E	flat forestland	1246	17	1304	23.91	16.97	0.05	4.86	61.39	17.19	1.53	25.76	0.3
14	China	31°54'N, 120°15'E	grassland	1200	16	1400	0.64	9.84	0.13	7.8	25.6	18.29	0.61	18.05	0.32
15	China	31°54'N, 120°15'E	flat forestland	1164	17	1841	1.97	20.72	7.91	7.15	76.6	25.48	2.28	28.15	0.33
16	China	31°54'N, 120°15'E	farmland	1164	17	1841	1.44	11.93	0.19	7.22	56.7	17.54	0.6	30.79	0.25
17	China	31°54'N, 120°15'E	farmland	1164	17	1841	1.63	20.7	0.19	7.4	37.7	18.8	1.09	15.57	0.32
18	USA	33°32'N, 121°52'W	farmland	1056	12	2200	2.91	9.85	0.05	5.2	4.65	15.3	0.99	11.93	0.23
19	Pakistan	33°04'N, 73°08'E	grassland	1143	27	3000	0.2	3.25	0.63	7.6	0.02	12.62	0.39	17.46	0.24
20	China	34°40'N, 112°21'E	farmland	708	14	2184	4.76	19.04	0.61	5.99	52.3	12.83	2.55	21.46	0.33
21	China	36°50'N, 115°49'E	farmland	528	13	2488	2.25	4.2	0.66	7.9	69.5	22.87	2.23	32.67	0.23
22	USA	38°13'N, 121°52'W	grassland	965	12	2200	3.84	5.91	0.03	3.47	3.25	12.28	1.07	10.62	0.24

23	USA	39°38'N, 121°15'W	flat forestland	965	12	2200	1.51	2.92	0.05	4.8	4	11.05	2.67	55.08	1
24	China	39°59'N, 116°10'E	flat forestland	593	12	2661	4.35	17.71	0.46	7.03	36	14.15	1.25	25.96	0.3
25	China	42°54'N, 129°28'E	farmland	518	5	2319	2.18	14.22	0.24	6.65	94.6	20.86	2.4	41.09	0.69
26	China	42°54'N, 129°28'E	farmland	518	5	2319	1.37	17.42	0.21	5.83	37.25	18.53	1.61	23.46	0.25
27	China	43°46'N, 87°40'E	farmland	73	8	3055	0.99	6.4	0.27	8.13	55.5	14.39	1.53	24.76	0.55
28	Italy	45°28'N, 9°10'E	grassland	1000	14	1400	2.21	10.5	0.08	6.59	5.3	22.26	2.82	47.96	1.1
29	Italy	45°28'N, 9°10'E	flat forestland	1000	14	1400	1.05	14.48	0.04	4.6	3.31	19.51	0.65	12.52	0.23
30	Italy	45°28'N, 9°10'E	farmland	1000	14	1400	3.05	21	0.05	5.05	3.14	16.62	1.77	24.22	0.69
31	Germany	53°33'N, 8°35'E	grassland	1000	11	2000	5.04	10.61	0.05	3.92	7.75	19.6	4.1	79.64	1.28
**WETLAND**															
1	Cameroon	4°37'N, 9°28'E	lake	2000	24	1825	22.48	1.12	0.18	5.03	3.3	17.3	3.45	18.46	0.25
2	Cameroon	4°37'N, 9°28'E	river	1246	17	1304	5.08	0.43	0.1	5.41	27.2	22.09	0.45	43.67	1.43
3	Cameroon	4°37'N, 9°28'E	river	1143	27	3000	9.71	1.12	0.75	7.73	0.03	32.1	0.78	13.5	0.87

4	Peru	12°06'S, 76°55'W	natural wetland	1000	19	3800	0.86	11.34	0.46	7.34	0.03	22.82	0.72	9.48	3.76
5	China	22°16'N, 113°34'E	estuary	1743	22	1722	161.13	19.45	0.27	7.56	73.4	40.37	1.96	32.98	3.71
6	Australia	27°34'S, 153°02'E	lake	4500	26	2737	0.62	1.13	0.39	5.19	0.02	2.61	1.34	19	0.27
7	Australia	27°34'S, 153°02'E	lake	4500	26	2737	1.63	0.22	0.03	9.03	0.67	38.32	0.23	1.83	0.45
8	China	29°20'N, 113°05'E	lake	1326	17	1741	43.52	4.54	0.35	7.77	41.8	36.18	0.93	10.83	5.52
9	China	29°30'N, 106°30'E	paddy field	1200	21	2920	20.14	2.3	0.37	5.62	0.17	27.35	0.57	10.63	0.29
10	China	30°44'N, 111°16'E	river	1200	21	2920	32.17	1.43	0.49	5.77	0.25	27.95	0.72	6.53	0.19
11	China	30°46'N, 120°42'E	paddy field	1100	18	1800	71.08	1.92	0.11	5.76	10.4	28.25	0.46	6.7	0.57
12	China	30°46'N, 120°42'E	wetland	1169	16	2017	53.2	4.34	0.02	8.02	23.86	26.55	0.84	9.9	0.28
13	China	31°31'N, 117°33'E	river	16	15	2942	6.48	0.8	0.15	8.54	2.06	31.83	0.73	12.07	0.69
14	China	31°31'N, 117°33'E	lake	1054	16	1978	184.58	18.15	0.07	6.93	14.58	30.41	1.3	9.08	0.77
15	China	31°31'N, 117°33'E	lake	1054	16	1978	6.04	17.88	0.14	4.82	48.1	14.12	0.55	10.65	0.29
16	Pakistan	33°04'N, 73°08'E	paddy field	1169	16	2017	45.33	16.37	0.55	7.78	0.02	31.09	0.96	22.87	0.29

17	China	34°20'N, 71°39'E	river	42	11	3147	67.08	20.5	10.07	6.65	44.49	25.32	3.08	32.61	1.89
18	China	34°38'N, 115°58'E	lake	708	14	2184	16.73	2.05	0.24	8.45	5.02	43.05	0.38	32.89	0.38
19	China	36°59'N, 119°E	river	319	10	2503	78.52	10.2	0.18	7.66	15.62	34.38	1.24	24.12	1.79
20	USA	38°06'N, 121°39'W	natural wetland	965	12	2200	36.76	1.57	0.25	7.87	47.46	42.03	4.76	44.41	2.33
21	USA	38°32'N, 121°52'W	lake	965	12	2200	3.55	2.97	0.05	4.53	2.3	19.73	0.82	11.74	0.64
22	USA	38°41'N, 121°52'W	lake	965	12	2200	42.34	2.68	0.05	4.42	3.85	35.97	0.52	10.21	0.25
23	China	38°54'N, 115°56'E	lake	528	13	2488	21.56	2.77	0.07	7.1	66.1	29.68	0.56	23.15	2.1
24	China	39°52'N, 117°25'E	river	529	10	2470	55.59	6.27	0.12	7.84	84.11	33.99	2.98	41.02	4.32
25	China	39°59'N, 116°10'E	constructed wetland	593	12	2661	7.15	0.21	0.26	6.4	94	54.6	1.89	34.82	0.86
26	China	39°59'N, 116°10'E	lake	629	13	2662	66.08	3.78	0.13	8.02	16.51	43.18	0.99	17.66	1.14
27	China	40°04'N, 116°31'E	river	625	12	2750	10.14	5.45	1.22	8.1	10.2	27.59	0.4	16.93	0.26
28	China	40°39'N, 121°25'E	natural wetland	645	8	2726	52.6	4.23	0.87	8.36	6.65	43.73	1.9	8.55	0.26
29	China	40°53'N, 108°54'E	lake	207	7	3202	146.62	17.55	0.14	8.35	49.73	28.57	2.2	39.3	11.15

30	China	43°48'N, 88°24'E	lake	594	2	2549	0.81	0.36	0.04	7.66	25.52	39.94	1.52	14.36	0.73
31	Italy	45°28'N, 9°10'E	river	1000	14	1400	9.24	3.13	21.13	5.58	9.63	27.1	4.47	71.88	1.23

The information of regional-scale factors (PRE: precipitation, T: temperature and SD: sunshine duration) is presented as an annual average value. The basic geographic information in each site was collected from the climate database of NOAA (National Oceanic and Atmospheric Administration, USA) (https://www.ncdc.noaa.gov/climate-information) and Wikipedia encyclopedia (https://www.wikipedia.org/). The information of local scale factors was measured after sampling during October 2015 to January 2016.

Supplementary Table 2 Sampling sites and background information in groundwater aquifers & snow

Country		Coordinate	Habitat Types	local-scale factors								
GROUNWATER				NH_4^+ (mg kg^{-1})	NO_3^- (mg kg^{-1})	NO_2^- (mg kg^{-1})	pH	TOM (%)	MC (%)	TN (g kg^{-1})	TC (g kg^{-1})	TS (g kg^{-1})
1	Australia	27°34'S, 153°02'E	groundwater aquifer	0.50	9.40	0.12	6.00	4.87	30.46	1.19	23.37	0.41
2	China TJ	39°52'N, 117°25'E	groundwater aquifer	15.59	8.27	0.12	7.84	84.11	33.99	2.98	41.02	4.32
3	China AT	42°54' N, 129°28'E	groundwater aquifer	1.56	4.58	0.03	6.58	2.63	21.69	0.74	8.63	0.16
4	Switzerland	46°56'N, 7°26'E	groundwater aquifer	0.66	1.02	0.06	8.62	1.2	36.23	0.23	104.99	0.17
5	Germany	53°33'N, 8°35'E	groundwater aquifer	1.99	4.19	0.12	6.34	2.07	18.34	1.04	26.11	3.09

6	Germany	53°33'N, 8°35'E	groundwater aquifer	2.22	2.43	0.05	6.43	1.95	18.76	0.88	19.34	2.35
7	China	28°13'N, 112°55'E	snow	5.00	4.58	0.03	4.81	ND	ND	ND	ND	ND
8	China	31°48'N, 117°13'E	snow	1.23	1.44	0.05	7.86	ND	ND	ND	ND	ND
9	China	31°47'N, 119°14'E	snow	5.78	5.38	0.11	7.4	ND	ND	ND	ND	ND
10	China	39°59'N, 116°10'E	snow	0.64	0.87	0.21	7.71	ND	ND	ND	ND	ND
11	China	42°16'N, 118°55'E	snow	0.05	0.07	0.03	7.98	ND	ND	ND	ND	ND
12	China	43°33'N, 87°42'E	snow	1.48	0.57	0.26	7.48	ND	ND	ND	ND	ND

The information of regional-scale factors (PRE: precipitation, T: temperature and SD: sunshine duration) is presented as an annual average value. The basic geographic information in each site was collected from the climate database of NOAA (National Oceanic and Atmospheric Administration, USA) (https://www.ncdc.noaa.gov/climate-information) and Wikipedia encyclopedia (https://www.wikipedia.org/). The information of local scale factors was measured after sampling during October 2015 to January 2016.

Supplementary Table 3 Sequences information of each ecosystem

Ecosystems	Samples number	Sequences number	OTUs number	Coverage
Dryland	31	18764-19997	32-67	99.84% - 99.95%
Wetland	31	18219-19987	26-63	99.82% - 99.97%
Groundwater aquifer	6	19393-19928	41-47	99.89% - 99.91%
Snow	6	19585-19999	3-17	99.95% - 99.99%

a. OTUs with relative abundance of more than 1% were calculated
